# Cancer testis antigen PASD1 expression and immunogenicity in human colorectal cancer and polyps

**DOI:** 10.55730/1300-0152.2623

**Published:** 2022-07-14

**Authors:** Nur Fazilah SUKOR, Azyani YAHAYA, Ismail SAGAP, Rahman JAMAL, Nor Adzimah JOHDI

**Affiliations:** 1 UKM Medical Molecular Biology Institute (UMBI), National University Malaysia, Cheras, Malaysia; 2 Department of Pathology, Faculty of Medicine, National University Malaysia, Cheras, Malaysia; 3 Department of Surgery, Faculty of Medicine, National University Malaysia, Cheras, Malaysia

**Keywords:** Colorectal cancer, polyps, immunotherapy, cancer testis antigen, PASD1, CD4 T cell response

## Abstract

Colorectal cancer (CRC) is a malignant tumor arising from a human inner colon lining that may spread to other organs such as the liver and lungs. Per ARNT Sim domain containing 1 (PASD1) is a cancer-testis antigen expressed in cancers including CRC but not in normal tissues except for normal testes. This study aims to study PASD1 protein as a potential target for CRC immunotherapy. A total of 90 CRC and polyps tissue samples were investigated for PASD1 RNA and protein expression using a real-time polymerase chain reaction and immunohistochemical staining, respectively. Matched patients’ peripheral blood mononuclear cells were pulsed with PASD1 peptides and measured for immunogenicity, cell cytotoxicity, and cytokine assays. The clinical data were collected and analyzed accordingly. Our results show that *PASD1_v2* mRNA expression was highly expressed in CRC (46.0%) and polyps samples (33.3%). Both PASD1-1 and PASD1-2 proteins were expressed in 31.7% of CRC and 29.4% of polyps samples. Protein expression was weak to moderate positive in the cytoplasm and/or nucleus of the tissues. Immune responses towards CD4-specific PASD1 peptides were detected in 21.7% of CRC and 23.5% of polyps patients. The most immunogenic peptide was PASD1 (1) in CRC while PASD1 (3) in polyps. Cytotoxicity effects were detected up to 57.20% observed in CRC samples while IL-17A and IL-6 cytokines were highly expressed. The demographic data suggest that Chinese female patients more than 60 years old, diagnosed with late-stage rectosigmoid tumors may benefit from the PASD1 peptide immunotherapy approach. This is the first report describing CD4-positive T-helper response to the PASD1 positive CRC patients and its cytotoxicity.

## 1. Introduction

Colorectal cancer (CRC) is a malignant tumor arising from a human inner colon lining that may spread to other organs such as the liver and lungs. It is the third most common cancer worldwide after lung and breast cancer with 1,931,590 total new cases recorded in 2020 and a mortality of 935,173 total cases (Bray et al., 2018; Azizah et al., 2019). Current standard treatments for CRC include surgery, chemotherapy and radiation therapy. However, 30%–40% of CRC patients who underwent these treatments experience tumor recurrence within 5 years of diagnosis. Due to the unspecificity of chemotherapy and radiotherapy treatments, several mild to severe side effects in patients such as massive loss of hair, persistent vomiting, frequent diarrhea and prolonged fatigue were observed (Schirrmacher, 2019). More specific and well-tolerated treatment alternatives are needed in improving the quality of cancer treatments. One of the options is targeted immunotherapy. Immunotherapy is a biological treatment that can naturally stimulate patients’ immune systems to specifically target and eradicate cancer cells with lesser side effects (Johdi and Sukor, 2020).

Cancer-testis antigen (CTA) is one of the attractive and widely studied immunotherapeutic targets due to its unique expression in various types of cancer cells but is absent in normal cells except testis tissue (Caballero and Chen, 2009). One of the CTAs is Per ARNT SIM domain containing repressor 1 (PASD1). PASD1 (other names: CT63, CT64, OXTES1) is a novel protein-coding gene that encodes transcription factor proteins with a PAS domain, putative leucine zipper and nuclear localization signal domains within the protein (Liggins et al., 2004). The PASD1 is located at Xq28 with genomic coordinates of GRCh38.p12 (151563184-151676739) in the human chromosome (Caballero and Chen, 2009). The PASD1 function as a biological clock suppressor which regulates the daily circadian rhythms of cells in humans (Michael et al., 2015). PASD1 genomic structure consists of two transcript variants of PASD1_v1 and PASD1_v2 due to the alternative splicing (Liggins et al., 2004). The longer PASD1_v1 transcript consists of 16 exons and a retained intron located between exons 14 and 15. The presence of stop codon (TGA) in the retained intron produced a shorter translated PASD1-1 protein of 639 amino acid (aa) from this PASD1_v1 transcript. The second transcript PASD1_v2 translates a 773 aa PASD1-2 protein with a unique C terminus region. Both of the PASD1 proteins consist of two overlapping PAS domains at the N terminal region of 32–94 aa and 41–137 aa (Liggins et al., 2004). PASD1 protein expression in normal testis tissues was detected in the nucleus and cytoplasm of primary spermatogonia at testicular tubules near the basal membrane which are restricted within the earliest phase of spermatogenesis (Liggins et al., 2004; Cooper et al., 2006). Its restricted expression patterns within the human gonads and various cancer cells make it one of the attractive immunotherapeutic targets. This study aims to validate PASD1 expression as a potential target for colorectal cancer immunotherapy by investigating its expression, immunogenicity of PASD1 peptide in inducing CD4 T cell immune response, cytotoxicity and cytokine expression. The results obtained were correlated with patients’ demographics. The study used a peptide-based cancer vaccine immunotherapy strategy. This type of approach is easy to synthesize, economical and more effective compared to other available options.

## 2. Methods

### 2.1. Ethical clearance

Ethical approval and written consent were applied according to the standard procedures of The Universiti Kebangsaan Malaysia Research Ethics Committee. All blood and tissue samples used in this study were collected under the rules and regulations stated. Reference number: UKM PPI/111/8/JEP-2016-063.

### 2.2. Samples selection

A total of 90 participants who came to the Endoscopy Service Centre, Department of Surgery, UKM Medical Centre from April 2018 to October 2019 and were subjected to perform full colonoscopy procedures were randomly approached and recruited in this study. Patients were assessed and consent was obtained from all participants before sample collection. There were 60 CRC patients, 17 colorectal polyps patients and 13 healthy controls participants. All samples were verified by the endoscopy specialist and histological pathologist.

### 2.3. Peripheral blood mononuclear cell (PBMC)

PBMCs are specialized immune cells that work together to protect our body from harmful pathogens. They are developed in the bone marrow from hematopoietic stem cells (HSCs). The three main cell categories present in PBMC composition include lymphocytes, monocytes and dendritic cells. PBMCs are collected by isolation from whole blood samples. In this study, 10 mL of fresh blood were collected from each patient in BD Vacutainer heparin tubes (Beckton Dickinson, USA). Samples were kept at 4 °C before further PBMC isolation. Samples were processed within 24 h postcollection. PBMCs were isolated with Ficoll Paque PLUS density gradient media (GE Healthcare, USA) according to the manufacturer’s protocol. Cells were cryopreserved in 10% of dimethyl sulfoxide solution (DMSO) in a liquid nitrogen tank (−196 °C) until use.

### 2.4. Cell lines and the cell culture

The SW480 and HCT116 CRC cell lines were purchased from the American Type Culture Collection (ATCC, USA). The cell lines were grown in Gibco Roswell Park Memorial Institute 1640 (RPMI-1640) culture media (Life Technologies, USA) supplemented with 10% heat-inactivated Gibco fetal bovine serum (FBS) (Life Technologies, USA) at 37 ºC with 5% carbon dioxide (CO_2_) concentration. The adherent cell lines were detached using Gibco 0.05% trypsin-EDTA (Life Technologies, USA) dissociation agent, washed with 1 X PBS and centrifuged for 5 min at 100 X g. The supernatant was removed after centrifugation and the pellet was resuspended in 1 mL of media before cells counting.

### 2.5. Real-time quantitative reverse transcription PCR (qRT-PCR) analysis

Total RNA was extracted from formalin-fixed paraffin-embedded (FFPE) sections using RNeasy FFPE Kit (QIAGEN, Germany) as per the manufacturer’s instruction. The thickness for each FFPE section was up to 20 μm per section. RNA extraction from cell lines was performed by using the RNeasy Mini Kit (QIAGEN, Germany) as per the manufacturer’s protocol. The extracted nucleic acid (RNA) was quantified for concentration and purity using a NanoDrop ND-2000C spectrophotometer (Thermo Scientific, USA). Samples with RNA concentrations of more than 10 ng/μL and purity A260/A280 within 1.70 to 2.10 were chosen for qRT-PCR analysis.

PASD1 has 2 different transcripts; PASD1_v1 and PASD1_v2. The qRT-PCR was performed at three different target sites. Primer A amplified the common region presented on both PASD1_v1 and PASD1_v2 transcripts. Primer A (F): 5′-TAC AGG AGC GGA AGA AGT GG-3′; Primer A (R): 5′-ACA GGA ACA ATG GGT TGG G-3′. Primer B amplified the end region of the PASD1_v1 transcript. Primer B (F): 5′-GCC CAC AAA CCT TGA AAT CAC-3′; Primer B (R) 5′-TCA CAC TCA CTT CCC TCT TAC-3′. The unique region on the longer PASD1_v2 transcript was amplified by Primer C. Primer C (F): 5′-AGC AGA CCA GAT TGA TGC C-3′; Primer C (R): 5′-CTA CCC ACT AAA CCC TAC CC-3′. Housekeeping gene control was GAPDH. GAPDH (F): 5′-CTG ACT TCA ACA GAG ACA CC-3′; GAPDH (R): 5′-TAG CCA AAT TCG TTG TCA TAC C-3′. All primers were designed using Primer-BLAST. The primers had 50%–60% GC content, and 19 to 22 base pairs with 58 to 61 °C melting points.

The qRT-PCR was performed by using Power SYBR Green RNA-to-CT 1-Step Kit (Applied Biosystems, USA). The total volume for each reaction is 20 μL. The reaction was performed using the standard thermal cycling conditions as recommended by the manufacturer. Triplicates samples were run in the Applied Biosystems 7500 Fast Real-time PCR System machine (Applied Biosystems, USA) and the C_t_ value was quantified.

### 2.6. Staining antibodies

Immunohistochemistry (IHC) was performed using the in-house PASD1a and PASD1b monoclonal antibodies (a kind gift from Dr. Kamel Ait-Tahar and his team at the Nuffield Division of Clinical Laboratory Science, University of Oxford). PASD1a (clone 2ALCC136) binds to the epitopes of PASD1-1 and PASD1-2 proteins (195–474 aa) within the common region of the N terminus on both transcripts and its localization is in the nucleus and cytoplasm of the cells. On the other hand, PASD1b binds to the unique C terminus region that is only present on PASD1-2 protein (540–773 aa) and its localization is within the cell nucleus (Cooper et al., 2006). Normal human testis and tonsil tissues were used as the positive and negative biological controls respectively.

### 2.7. The formalin-fixed paraffin-embedded (FFPE) tissue block

FFPE blocks from CRC and polyps patients were obtained from the Department of Pathology, UKMMC and validated by the pathologist. Only blocks with more than 80% tumor tissues were selected for IHC staining. The blocks were sectioned at 5 μm using Leica RM2245 semiautomated rotary microtome (Leica Biosystems, Germany) and processed using a standard sectioning protocol.

### 2.8. Immunohistochemical staining

IHC staining was performed using EnVision FLEX Mini Kit, High pH, Dako Autostainer/Autostainer Plus (Dako, Denmark) as per the manufacturer’s instructions. Tissue sections were incubated with primary antibodies of PASD1-1 (clone 2ALCC136) and PASD1-2 (clone 2ALCC128) at final concentration of 10 μg/mL for 20 min. Anti-PASD1 antibodies were treated with horseradish peroxidase immunoglobulins (HRP) secondary antibody and detected with 3, 3′-diaminobenzidine (DAB) substrate.

### 2.9. CD4 PASD1 peptides

All PASD1 peptides were generated using TEPITOPE and SYFPEITHI prediction algorithm software as described before (Ait-Tahar et al., 2009; Ait-Tahar et al., 2011). The peptides were synthesized through standard chemistry on a multiple peptide synthesizer and were more than 90% pure (a kind gift from Dr. Kamel Ait-Tahar and his team at Nuffield Division of Clinical Laboratory Science, University of Oxford). The peptides are listed in Table 1. Lyophilized peptides were diluted in DMSO and stored at −20 °C until further usage.

### 2.10. ELISpot assay

ELISpot assay was performed using the Human Interferon-gamma (IFN-γ) ELISpot^Basic^ HRP Kit (Mabtech, Sweden) according to the manufacturer’s instruction. The PBMCs were pulsed with different peptides [PHA, irCD4, PASD1 (1), PASD1 (2) and PASD1 (3)] at a concentration of 10 μM. After 7 days, PBMC was restimulated with the respective 10 μM peptides and the cell suspension was transferred into the antihuman IFN-γ coated PVDF plate. Signals were detected with biotinylated 7-B6-1 detection antibody (Mabtech, Sweden) and streptavidin-horseradish peroxidase (HRP) (Mabtech, Sweden) as per the manufacturer’s recommendation. The number of spots formation was quantitated using ImmunoSpot S6 Universal Elispot/Flourospot Analyser (Cellular Technology Limited, USA). Each spot represents a positive IFN-γ response towards the peptides.

### 2.11. T cell expansion

CRC or polyps samples with high IFN-γ response towards PASD1 peptide were selected for further T cell expansion to generate a T cell line. Cells were cultured in Gibco CTS OpTmizer T-cell Expansion SFM media (Life Technologies, USA) and incubated at 37 ºC in 5% CO_2_. After 72 h, an equal volume of CTS OpTmizer T-cell Expansion SFM containing 50 IU/mL of human recombinant interleukin of IL-2 was added to each well of the 96-well plates. Cell media was replaced with a fresh medium every 3 days and cells were restimulated with PASD1 peptide weekly for up to 5 weeks.

### 2.12. Cytotoxicity assay

SW480 (PASD1-positive) and HCT116 (PASD1-negative) target cell lines were cultured as described before and stained with 0.1 μM BD Pharmingen Calcein AM (BD Bioscience, USA) for 30 min at 37 °C and protected from light. The generated patients’ T cell lines were added to the stained target cells with an effector: target ratio of 10:1, 20:1 and 40:1. Cells were cocultured for 18 h at 37 °C in 5% CO_2_. Next, the cocultured cells were labelled with PerCP-Cy 5.5 mouse antihuman CD3 (BD Bioscience, USA), 5 uL of APC Annexin V (Annexin V) (BD Bioscience, USA) and Propidium Iodide (PI) staining solution (BD Bioscience, USA) as per manufacturer’s protocol.

### 2.13. Cytokine profiling

Cytokine profiling was performed using the BD Cytometric bead array (CBA) human Th1/Th2/Th17 cytokine kit (BD Bioscience, USA) manufacturer’s instruction. The list of cytokines detected were interleukin-2 (IL-2), interleukin-4 (IL-4), interleukin-6 (IL-6), interleukin-10 (IL-10), tumor necrosis factor (TNF), interferon-gamma (IFN-γ) and interleukin-17A (IL-17A). Cytokine expression was quantified with BD FACSVerse flow cytometer (BD Bioscience, USA). Results were analyzed using FCAP Array v3.0 software (BD Bioscience, USA).

### 2.14. Statistical and correlation analysis

The qPCR, ELISpot, apoptosis and cytokine assays results were analyzed using the Student’s t-test. A statistics of *p*-value that measures the probability of obtaining the observed results, assuming that the null hypothesis is true used for analysis. The lower the p-value, the greater the statistical significance of the observed difference. The *p*-values < 0.05 were considered significant. Correlation analysis performed on the patients’ demographic data with PASD1 expression and CD4 T cell response data was based on a prospective frequency basis.

## 3. Results

### 3.1. Patients demographic data

A total of 90 samples from 3 different groups of patients; colorectal cancer (CRC) (n = 60), polyps (n = 17) and normal controls (n = 13) were used in this study (Supplementary S1). The median age of the CRC, polyps and normal controls was around 66 years old. Higher male respondents with an age >60 years old were observed. Approximately 46.7% of CRC samples were from Malay and Chinese patients while 58.8% of polyps samples and 53.8% of the normal control samples were Chinese. Most of the CRC patients (83.3%) had a tumor at the left side (distal) part of the colon which includes anal, rectum, rectosigmoid, sigmoid, descending colon and splenic flexure compared to the right side (proximal). About 41.7% of these patients were diagnosed at stage III, indicating cancer has invaded from muscularis propria into pericolorectal tissue. Out of 61.7% who presented with nodal metastasis CRC, only 20.0% of patients were diagnosed with distant metastasis. Adenocarcinoma which consists of mucinous and synchronous adenocarcinoma was the commonest type of CRC in this study (98.3%). Similar to the CRC samples, the polyps were also located at the left-sided colon (64.7%) with the majority being from hyperplastic polyps.

### 3.2. PASD1 mRNA expression

#### 3.2.1. PASD1 mRNA expression in CRC cell lines

**Figure 1:**
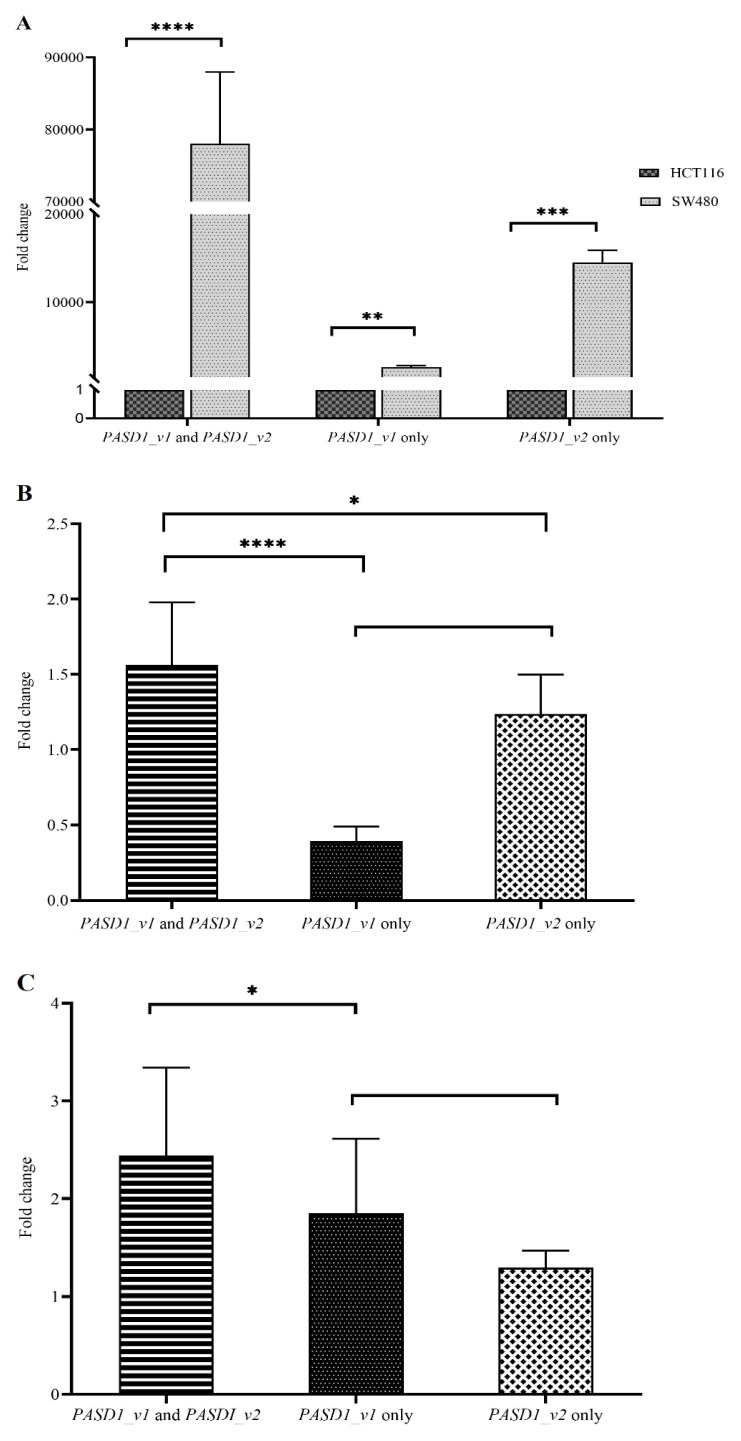
PASD1 mRNA expression in (A) SW480 and HCT116, (B) CRC and (C) polyps samples. Student t-test with * p ≤ 0.05, **p ≤ 0.01, ***p ≤ 0.001 and p ≤ 0.0001. Error bar indicates mean with SEM.

PASD1 mRNA was highly expressed in the SW480 CRC cell line while no PASD1 mRNA was detected in the HCT116 CRC cell line (Figure 1a). The common region of PASD1_v1 and PASD1_v2 mRNA in SW480 cells showed the highest expression of around 78,000-fold change. Of these two transcripts, PASD1_v2 expression was higher. The SW480 cell line was selected as a PASD1 positive cell line while HCT116 was used as a negative control cell line throughout the experiment.

#### 3.2.2. PASD1 mRNA expression in clinical samples

A total of 50 out of 60 CRC and 6 out of 17 polyps FFPE blocks sections were available for qRT-PCR analysis. These FFPE blocks matched with the ones used for immunohistochemistry analysis (subsection 3.3). In other words, 10 CRC and 11 FFPE blocks were not sufficient for qRT-PCR analysis.

In CRC samples, a total of 14 out of 50 patients expressed both the PASD1_v1 and PASD1_v2 transcripts (Table 2). Among the CRC samples, PASD1_v2 was also frequently expressed (46%) compared to PASD1_v1 (10%). The expression level of both PASD1_v1 and PASD1_v2 was the highest with 2-fold relative expression compared to the PASD1_v1 only and PASD1_v2 only (Figure 1b). The relative PASD1 expression for PASD1_v2 was also higher compared to the PASD1_v1 transcript by around 1-fold difference. In polyps samples, approximately 33% (2 out of 6) were PASD1_v1 and PASD1_v2 transcripts positive (Table 2). The same patients were also presented with PASD1_v1 only or PASD1_v2 only. Contrary to the SW480 cell line and CRC sample, PASD1_v1 was expressed 0.5-fold higher than PASD1_v2 in polyps patients (Figure 1c). Overall, PASD1 mRNA expression was higher in the polyps group compared to the CRC with 1-fold difference.

### 3.3. PASD1 protein expression

**Figure 2:**
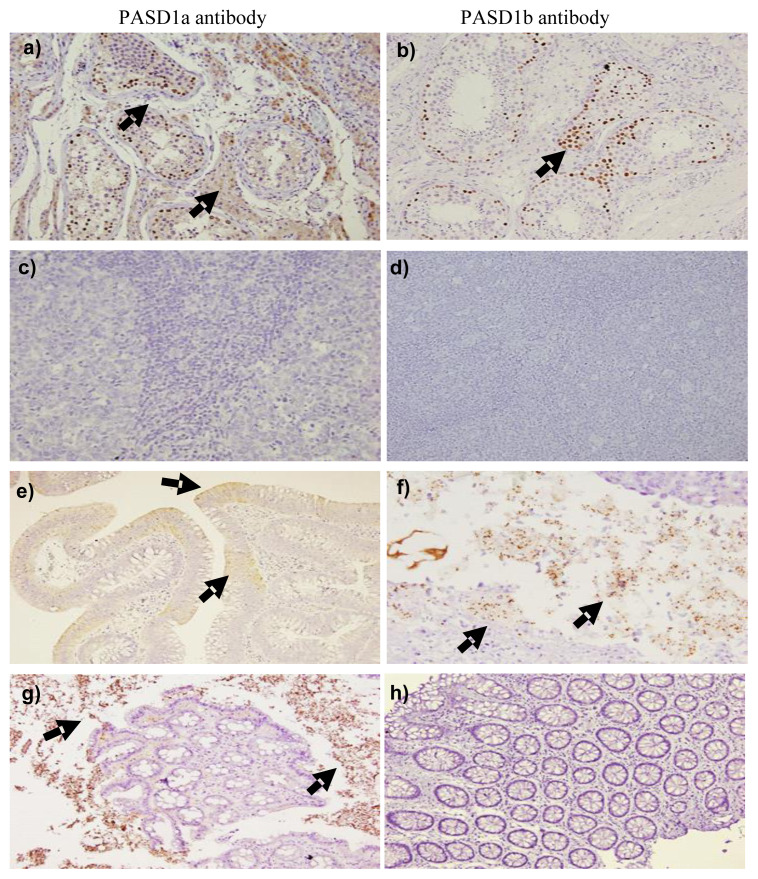
Representative IHC staining of the human tissues with PASD1a antibody (the left column) and PASD1b antibody (right column). A) Normal testis tissue (positive control) stained with PASD1a antibody. B) Normal testis tissue (positive control) stained with PASD1b antibody. C) Normal tonsil tissue (negative control) stained with PASD1a antibody. D) Normal tonsil tissue (negative control) stained with PASD1b antibody. E) CRC tissue stained with PASD1a antibody. F) CRC tissue stained with PASD1b antibody. G) Polyps tissue stained with PASD1a antibody. H) Polyps tissue stained with PASD1b antibody. Black arrows refer to the brown staining in the nucleus and/or cytoplasm of the cells, suggesting positive PASD1-1 and/or PASD1-2 protein expressions. No brown staining was observed within the nucleus and/or cytoplasm of the tissues of C, D and H suggesting no expression of these proteins. Photos were taken under 200× magnification.

PASD1-1 and PASD1-2 protein staining was carried out using PASD1a and PASD1b antibodies, respectively. Positive staining with the PASD1a antibody showed strong brown staining in the nucleus and cytoplasm of the tissue while staining with the PASD1b antibody showed strong staining in the nucleus with no staining in the cytoplasm (Figure 2).

#### 3.3.1. PASD1 protein expression in CRC

A total of 60 CRC and 17 polyps FFPE blocks sections were analyzed for immunohistochemistry. CRC tissues showed moderate staining with both the PASD1a and PASD1b antibodies. For the PASD1a antibody, staining was mostly in the nucleus and cytoplasm of the lamina propria and muscularis mucosa area of the colon tissue while staining with PASD1b antibody was in the nucleus of the lamina propria and muscularis mucosa (Figures 2e and 2f). PASD1-1 and PASD1-2 protein were expressed at varying degrees and the frequency of this staining is summarized in Table 3. About 21.7% showed weak positive and 10.0% with moderate positive using PASD1a antibody and 3.3% were weakly stained with PASD1b antibody. The PASD1 positive CRC samples were mostly from patients aged ≥ 60 years old (63%) and female (63%) (Supplementary S2). Chinese patients were the highest frequency (58%) compared to other races. About 84.2% of CRC patients had their tumor in the distal colon and all of the patients performed surgical resection to remove cancer.

#### 3.3.2 PASD1 protein expression in polyps

The polyps showed weak staining with the PASD1a antibody (Figure 2g). Most of the positive stained polyps samples were weakly positive (23.5%) while 5.9% were stained with moderate positive (Table 3). No PASD1-2 protein was detected in polyps samples using the PASD1b antibody (Figure 2h). In both CRC and polyps tissues, PASD1-1 and PASD1-2 proteins were detected with higher frequency compared to PASD1-2 only protein. Similar to CRC samples, the PASD1 positive polyps samples were mostly from patients aged ≥60 years old (80%) but males showed higher frequency (80%) (Supplementary S3). All samples were from Chinese patients, 80% of the polyps were at the distal colon and all were hyperplastic polyps.

### 3.4 Interferon-γ (IFN-γ) release assay towards CD4 PASD1 peptides

Immunogenicity of the CD4 PASD1 peptides was carried out using an ELISpot assay. About 21.7% (13/60) CRC samples and 23.5% (4/17) polyps patients showed positive CD4 immune response with interferon-gamma (IFN-γ) secretion toward any of the PASD1 peptides (Table 4). Of these, 7 CRC samples (11.7%) showed IFN-γ release towards PASD1(1) peptide, 1 sample (1.7%) with PASD1(2) peptide and 5 samples (8.3%) with PASD1(3). In polyps, 1 sample (5.9%) secreted IFN-γ towards PASD1 (1) and PASD1 (2) peptides, respectively, and 2 out of 17 samples (11.8%) secreted IFN-γ towards PASD1 (3) peptide. PASD1(1) was the most immunogenic peptide in CRC samples while PASD1(3) was the most immunogenic peptide in polyps.

In the ELISpot assay, the results were considered positive only if the number of spots counted in either one of the PASD1 peptides was at least twice the number of spots presented in the control cultures of cells only (without any peptide) and the irrelevant CD4 peptide (irCD4) (Ait-Tahar et al., 2011). The irCD4 peptide was the peptide derived from HIV-1 reverse transcriptase with sequence DESFRKYTAFTIPSMNNETP. It was selected as the negative control peptide as it has a stable reverse transcriptase structure of heterodimer made up of 51-kDa and 66-kDa subunits and was less affected by heterodimerization binding. Thus, it served as a good negative control peptide for the PASD1 peptide in this study.

### 3.5. Cytotoxicity assay

**Figure 3:**
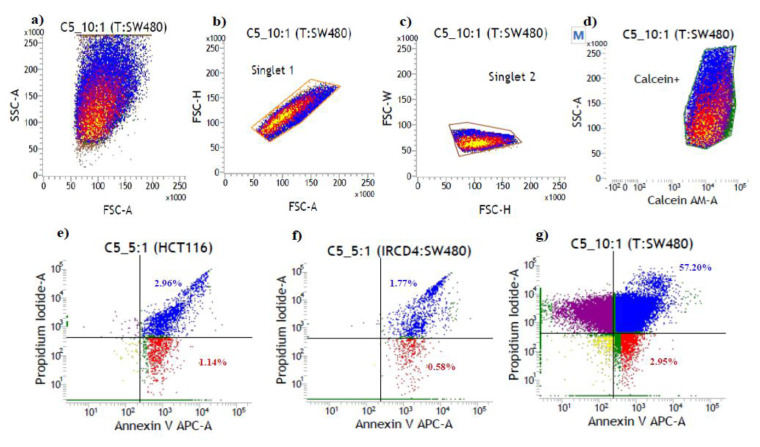
Immunophenotyping and apoptosis assay results for sample C5. a) The cocultured cells population based on its size and granularity, b) Singlet cells, c) Gated singlet cells quantitated, d) Live PASD1-positive SW480 cells population that was positively stained with calcein. Apoptotic cells quantitated in T cells cultured with e) PASD1-negative HCT116 cell line control, f) irCD4 peptide control, and g) SW480 PASD1-positive cell line. The blue color indicates the late apoptotic cells while the red color showed the early apoptotic cells. Live and necrotic cells were represented with yellow and purple colors respectively.

Based on availability and positive IFN-γ release, the T cells from samples C5, C12, C32, C6 from CRC samples and 1 normal healthy volunteer T cell were expanded in vitro and pulsed with their respective peptides based on the outcome from their response in the ELISpot assay. These T cells later were cocultured with SW480 and HCT116 CRC lines in a dose-dependent fashion as mentioned in the method before. The cytotoxicity effect was quantitated via apoptosis assay and analyzed using flow cytometry. The representative data is shown with sample C5 (Figure 3). Post 18 h of coculture, 57.20% of PASD1-positive SW480 cells showed late apoptosis and 2.95% of the cells were in early apoptosis (Figure 3g). No significant lysis was observed in the PASD1-negative HCT116 cell line (Figure 3e) and irCD4-cultured T cell line (Figure 3f). These controls showed a lower percentage of late and early apoptotic cells between 1% and 3%. Of the four CRC samples that were tested for cytotoxicity assay, only sample C5 showed a significant cytotoxicity effect on PASD1-positive SW480 cell lines and the T cells were stimulated against PASD1 (1) peptide. No significant cytotoxicity effects were observed in samples C6, C12, C32 and normal healthy control (data not shown). This study is the first to show data to suggest CD4 Th cell killing in the PASD1 positive SW480 cell lines via apoptosis.

### 3.6. Cytokine analysis

**Figure 4:**
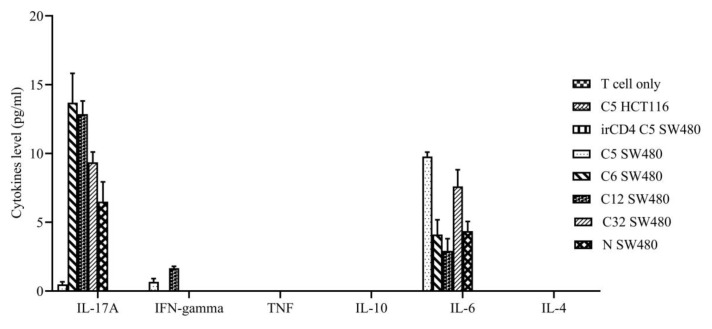
Cytokines level measured following 18 h of coculture. IL-17A and IL-6 were detected in all of the CRC and normal control samples cultured with a PASD1-positive SW480 cell line whereas only two samples (C5 and C12) released IFN-gamma. Some cells e.g., T cells only, C5 HCT116, irCD4 C5 SW480 did not show any detectable cytokine production, hence no bar chart for these respective samples. Error bar indicates mean with SEM.

Cytokine was quantified from the coculture cells consisting of CRC cell lines (SW480 and HCT116) and T cell lines generated from samples C5, C12, C32, C6 and normal healthy control. The cytokines measured were IL-17A, IFN-γ, TNF, IL-10, IL-6 and IL-4. IL-17A and IL-6 levels were the highest released during the co-culture while no TNF, IL-10 and IL-4 were detected in all of the samples (Figure 4). IL-17A was highest at 12.19 pg/ml in samples C12 and C6 while IL6 was detected between 6.75 and 10.00 pg/mL in samples C5 and C32. Only two samples (C5 and C12) released IFN-γ throughout the coculture (between 0.51 and 1.76 pg/mL).

### 3.7. Descriptive frequency correlation of patients’ demographic data with PASD1 expression and T cell response

Descriptive frequency correlation of the demographic data, PASD1 expression and immunogenicity of CRC and polyps samples towards PASD1 peptides were analyzed. Most of the patients were more than 60 years old, female and of the Chinese ethnicity. Cancer originated in the rectosigmoid part of the colon and patients were diagnosed at an advanced stage of stage T3 or T4. Positive PASD1-1 protein expression was also observed in all of these patients. Of these, only samples C5 and C9 showed positive PASD1 mRNA expression. In polyps samples, no correlation in PASD1 mRNA or protein expression was observed although all of the polyps samples elicited CD4 T cell response towards any of the PASD1 peptides.

## 4. Discussion

Out of the 60 CRC samples collected in this study from April 2018 to October 2019, 36 were from male patients and 24 from female patients. This sex proportion is consistent with the Malaysia National Cancer Registry Report (MNCRR 2012–2016), where a higher incidence of male CRC patients was recorded compared to the female (Azizah et al., 2019). It could be due to the sex-hormonal differences between males and females that affect CRC incidence and progression. Lower sex hormone-binding globulin (SHBG) and testosterone levels along with a higher proportion of estradiol over testosterone were correlated with a higher risk of developing CRC in males (Lin et al., 2013). As for the ethnicity, equivalent samples were collected from Malay and Chinese while 3.3% were from Indian and other ethnicities such as Punjabi and Kenyah. Hence, this was a reasonable representation of the Malaysian population as there was multiethnic participation in the study.

The median age in our sample population was 66.5 years old. About 68.3% of the total samples were patients with age more than 60 years old during diagnosis. This is in concordance with what was reported in MNCRR 2012–2016. The age-standardized rate (ASR) of patients aged 60 to 64 years old was 70 cases per 100,000 and the increasing pattern was shown in patients more than 75 years old (Azizah et al., 2019). One possible reason that contributes to this is the aging process that eventually reduced the lymphocytes’ production hence weakening the immune system function or known as immunosenescence (Giunco et al., 2019). Thus, precancerous and tumor cells were less recognized by the minimally produced immune cells. This evasion of immunosurveillance is also listed as one of the emerging hallmarks of cancer (Hanahan and Weinberg, 2011).

In terms of cancer staging, poor awareness regarding CRC early symptoms and risk factors might be the reason why most of our sample population were diagnosed at a late stage of T3 and T4. In a study that was conducted in the Klang Valley area, there were around 8.2% and 8.5% of respondents without any knowledge of early signs and risk factors of CRC, respectively, indicating there is still limited awareness among urban Malaysians about this disease (Sindhu et al., 2019). However, a recent study suggested that a factual-based mass media campaign on CRC can enhance awareness in over 65% of the Malaysian participants (Schliemann et al., 2020).

The majority (83.3%) of the tumors among CRC patients in this study were located in the distal (left-sided) part of the colon. However, there was no clear evidence on the relation between the location of the tumor and cancer prognosis due to contradictory findings in several studies (Bustamante-Lopez et al., 2019).

Almost all of the histological subtype of CRC in this study was from adenocarcinoma which consisted of classical and mucinous adenocarcinoma. This might be due to the nature of the CRC itself which originates from epithelial cells of the colorectal mucosa (Fleming et al., 2012). Only one of our samples showed properties of the rare neuroendocrine carcinoma (NEC) subtype and this patient was diagnosed with stage IV CRC. The polyps patients were males (70.5%). Our results supported the recent study conducted at Serdang Hospital which reported that the polyps detection rate is higher in males (22.5%) than in females (17.1%) (Tan et al., 2019). The protective function provided by the estrogen released causing reduced angiogenesis was postulated to have a crucial role in lowering the risk of colonic polyps formation among women (Yang et al., 2013). This might be one of the possible reasons why polyps detection rates were found higher in males compared to females. The polyps samples in this study were mostly hyperplastic polyps (70.5%). The hyperplastic polyp is a type of rarely malignant polyps whereas most sessile polyps are known as precancerous colonic lesions with indefinite shapes (Pai et al., 2019). The prevalence of colorectal polyp increased with age and a similar trend was also observed in the Chinese (Pan et al., 2020) and Thai populations (Siripongpreeda et al., 2016). This might be the result of both immune senescence and genetic instability resulting in cellular destruction (Vijg J et al., 2017). The healthy cells can continue to divide out of control and thus develop into colorectal polyps among the elderly group.

We observed a strong positive PASD1 mRNA expression in the SW480 CRC cell line and it was the only CRC cell line expressed with PASD1 mRNA. This finding is following previous reports (Liggins et al., 2004). SW480 is a Dukes’ B colorectal adenocarcinoma cell line. The reason for this remains elusive, however, SW480 also expresses other X-linked CTAs such as MAGEA2, MAGEA3, MAGEA6 and GAGE8 (Yamada et al., 2013). Thus, the SW480 cell line serves as a good positive control cell line model in several CTAs expression studies including PASD1.

The alternatively spliced variant PASD1_v2 showed the highest expression in CRC groups and 69.5% of CRC patients expressing PASD1_v2 were diagnosed at the late stage of T3 and T4, which often leads to a poor prognosis of the disease. This could suggest the potential role of PASD1_v2 in colorectal carcinogenesis and might serve as a favorable predictive marker of high-risk CRC patients. This supports a previous study on diffuse large B cell lymphoma (DLBCL) cell lines that showed the potential of PASD1_v2 as a subtyping marker in recognizing high-risk DLBCL patients as it was expressed only in poor prognosis nongerminal center subtype derived cell lines (Liggins et al., 2004). Interestingly, PASD1 mRNA expression was observed in many of the hyperplastic polyps samples instead of tubulovillous or tubular adenoma type. Hyperplastic polyps were reported to have lesser potential in neoplastic transformation compared to the other types of polyps due to the limited expression of cancer-related genes (Rahmatallah et al., 2017). To the best of our knowledge, the expression of PASD1 has not yet been reported in any types of colorectal polyps samples. Nevertheless, studies on the differentially expressed genes among unifocal colon polyp samples show that there were approximately 274 upregulated genes in colon polyps and they play a crucial role in immune response (Lian et al., 2015). Therefore, the expression of PASD1 in polyps samples could be further explored especially in terms of its biological function in the patients’ immune systems.

The IHC staining results on the human normal testis tissue (positive control for PASD1 expression) suggested that the common regions of PASD1-1 and PASD1-2 were expressed at the nuclei and cytoplasm while the unique PASD1-2 protein region was restricted only at the nucleus part of the primary spermatogonia cells near basal membrane in testicular tubules. The unique region of PASD1-2 might have some deficiency in the translational process that affects the transportation of the protein into the cytoplasm that needs further exploration. The testis organ is one of the immune privilege sites due to a lack of MHC class I expression (Scanlan et al., 2004). Therefore, targeting CTAs such as PASD1 may not lead to a catastrophically autoimmune response against patients’ own normal and germline tissues. This is imperative in minimizing the adverse effects of immunotherapy on normal healthy tissues in CRC patients. As expected with normal tissues, no PASD1 expression was observed in the human normal tonsil tissue (negative control for PASD1 expression, indicating the high specificity of the PASD1 antibodies in detecting the PASD1 protein expression. This study is also following previously published data on PASD1 protein expression and localization in the tissues (Cooper et al., 2006; Johdi et al., 2019). These findings show that PASD1 is indeed an ideal candidate for an immunotherapeutic target due to its expression specificity.

This current study shows the highest PASD1 protein expression in the CRC population (31.7%). A previous study described only 17.4% PASD1 expression in CRC (Soh et al., 2019). The possible reason for these discrepancies was the PASD1 antibodies used in both studies. In our study, the PASD1 antibodies were in-house monoclonal antibodies designed by the collaborators to specifically target the common and unique regions of PASD1 proteins (Cooper et al., 2006). PASD1a recognized 195 to 474 aa (a common region in both PASD1-1 and PASD1-2 proteins) and PASD1b recognized 540 to 773 aa, the unique region that only presented in the longer PASD1-2 protein. A previous study reported using a commercially available polyclonal PASD1 antibody targeting 301 to 600 aa (Thermo Fisher Scientific) (Soh et al., 2019). The differences in binding epitopes for PASD1 antibodies may result in disparities in PASD1 protein expression levels in CRC samples. Secondly, a bigger sample size increases the chances of detection for positive PASD1. Our study analyzed 60 CRC patients compared to only 23 CRC patients in the previous study [32]. Moreover, the staining reagents and materials also play a crucial role in protein detection. The kit used in our study, EnVision FLEX Mini kit, high pH, Dako autostainer/autostainer plus (Dako, Denmark) is a well-established kit that is optimized for IHC detection and commonly used in clinical and published papers.

We demonstrated a higher expression of PASD1-1 and PASD1-2 common region compared to the unique PASD1-2 protein. Similar findings were also observed in the PASD1 expression study in lymphoma particularly the DLBCL patients (Cooper et al., 2006; Johdi et al., 2019). This might due to the longer epitope coverage in the PASD1-1 and PASD1-2 common region which started from 195 to 474 aa while the unique region only covered from 540 to 773 aa (Cooper et al., 2006). Hence, a larger binding epitope was presented at the common region which eventually may increase the frequency and chances of antibody binding at that particular site. We also observed a low to moderate brown staining expression at the common region of PASD1-1 and PASD1-2 mainly at the lamina propria and muscularis mucosa of the late-stage CRC tissue. This could be due to the presence of invading cancer cells in several nodules and other neighboring organs. Besides CRC and polyps, PASD1 expressions have been reported in other cancers such as DLBCL and acute myeloid leukemia (AML) (Cooper et al., 2006) and glioma patients (Li et al., 2019) in which, all the studies involved cancer at an advanced stage. This shows that PASD1 might have a role in carcinogenesis and a potential subtyping marker in recognizing high-risk cancer.

Having said that, a low to moderate expression level of the common region PASD1-1 and PASD1-2 protein was also detected in polyps while no PASD1-2 protein from a unique region was detected although PASD1_v2 mRNA was expressed in some of the polyps samples. This can occur due to point mutation that may lead to a reduced protein expression in this region as shown in a study with MAGE-A4 where certain point mutations affect the protein expression and thermal stability of the protein (Hagiwara et al., 2016). Also, the absence of PASD1-2 protein expression in polyps that were detected in many cancers might be well correlated with the stage of the disease.

In general, we observed a higher PASD1 mRNA expression level compared to the protein level. The discrepancy in RNA and protein expression has been reported in several CTAs expression studies including in FATE-1 (Doghman-Bouguerra et al., 2020). This is due to epigenetic and nonepigenetic events. Epigenetic modifications such as DNA methylation could affect the CTAs expression and regulation mechanism in normal and cancer cells and induce promoter silencing mainly in the early stage of CRC, hence reducing the gene expression. This could explain why the PASD1 protein staining was mostly observed in stages 3 and 4 CRC patients. Other factors could be the posttranslational histone modification that may lead to the downregulation of PASD1 expression in CRC and polyps patients. Histone deacetylation process can lead to chromatin compaction thus inhibiting the binding of the transcription factors and RNA polymerase at the promoter region, and therefore results in limited gene expression.

Nonepigenetic events such as cytokine levels may also contribute to CTAs’ expression levels. As an example, Semenogelin 1 (SEMG1) expression is positively correlated with the presence of interleukins (IL-4 and IL-6) in multiple myeloma cells (Zhang et al., 2009). Interestingly, PASD1 upregulation was associated with the activation of signal transducer and activator of transcription 3 (STAT3). The STAT3 transcription factor is predominantly activated by the presence of the IL-6 cytokine. However, in our study, a low level of IL-6 and the absence of IL-4 cytokines were detected in the CRC samples. This might explain the low or no PASD1 expression detected in some of the CRC patients.

Also, the PASD1 protein expression could be below the detection level of the IHC technique compared to the highly sensitive method in qRT-PCR. The IHC technique highly depends on the antigen-antibody epitope binding. The translation process from mRNA to protein involved many factors (stability of the mRNA template, binding of each amino acid to the most suitable transfer RNA (tRNA), the specificity of the codon base pairing) as well as processes (initiation, elongation, termination) and it is not a straightforward process. Thus, disparities in mRNA and protein expressions were expected. Both techniques complement each other and are crucial in determining the overall landscape of PASD1 expression. However, the IHC technique is the most appropriate method to determine the PASD1 cellular localization in the tissue and possible secondary PASD1 isoform which is not within the detection range of currently available common or unique PASD1 epitopes (Ait-Tahar et al., 2011). Therefore, this may provide a better in-depth study avenue to improve the epitope target for PASD1 antibodies in the future.

The current study was also performed to identify short immunogenic PASD1 peptides capable of eliciting CD4 Th cell responses. Such peptides could then be included in a CRC vaccine immunotherapy as well as colorectal polyps expressing PASD1. Three potential CD4 Th PASD1 peptides of 8 amino acids in length were studied and all of them were able to elicit interferon-γ responses in some CRC and polyps patients, with the PASD1 (1) and PASD1 (3) peptides being identified as the most immunogenic peptides in CRC and polyps, respectively. PASD1 (1) peptide binds to an epitope at the common region of the PAS domain (38 to 47 aa) and is frequently found in most solid tumors (Cooper et al., 2006). This is possibly the reason for the higher detection frequency of PASD1 (1) peptides compared to the other peptides and has the potential to be further explored as a candidate for immunotherapy. The lack of significant homology of the PASD1 peptides with other molecules combined with the absence of any significant response with the irrelevant peptide and normal sample demonstrates the specificity of the response to PASD1.

The interferon-γ responses to the PASD1 peptides were present in 11 of the 60 CRC patients and 3 of the 17 polyps patients whose tumors expressed PASD1 protein. However, 2 CRC patients and 1 polyps patient recognized the PASD1 peptides but lacked detectable PASD1 protein. A disparity between PASD1 expression and immunogenicity has been described previously (Ait-Tahar et al., 2011) and it is possible that the immunolabeling techniques used here may not constitute a sufficiently sensitive tool to identify low levels of PASD1 protein expression. Importantly, high levels of target protein expression may not be necessary for immune recognition. To date, this is the first study describing the CD4 T cell response towards PASD1 peptides in CRC and colorectal polyps patients. Previous studies have reported on CD4 responses in DLBCL patients (Ait-Tahar et al., 2011).

Interestingly, all of the polyps samples that showed CD4 T cell response against PASD1 peptides were from hyperplastic polyps, a type of polyps with less association with cancer development (Rahmatallah et al., 2017). This is consistent with the PASD1 protein expression data. This could suggest that hyperplastic polyps do have malignant potential due to hypermethylation which affects their morphological features to transform into malignant cells and could be further elucidated.

We managed to raise CD4 Th cell lines from 4 CRC patients and 1 normal healthy volunteer. T cells from samples C5, C12 and C32 showed the highest response toward PASD1 (1) peptide while sample C6 exhibited the highest response towards PASD1 (2) peptide. The normal healthy sample was raised in PASD1 (1). Of these, only the C5 sample showed significant lysis of PASD1-positive SW480 tumor cell lines but not in the PASD1-negative HCT116 cell line, normal healthy sample and irCD4. This suggests that the CD4 T cells were able to recognize endogenously expressed PASD1 and specifically lyse PASD1-positive cell lines. The result is in agreement with data from a previous study on CD4 T cell response to PASD1 protein in DLBCL patients where a higher percentage of cell lysis was observed in the PASD1-positive Thiel cell line compared to the PASD1-negative OCI-Ly3 and SUDHL-6 cell line control (Ait-Tahar et al., 2011). However, further optimization may be required to obtain a good baseline in normal samples. We also did not observe significant apoptosis in the other 3 CRC samples although the samples produced high CD4 T cell response after stimulation with the PASD1 peptide in ELISpot.

Although CD8 cytotoxic T lymphocytes (CTLs) are the main cells involved in tumor cell killing, previous studies have reported the imperative roles of the CD4 T cells in orchestrating antitumor response (Ait-Tahar et al., 2011). The CD4+ T cells act as central coordinators by either suppressing or promoting the antitumor CTL response via effector cytokine secretion such as interferon-gamma and interleukin 17A. This provides evidence of measuring the CD4 immune response in understanding their role in antitumor response in CRC and polyps.

Interleukin 17A (IL-17A), interferon-gamma (IFN-γ) and interleukin 6 (IL-6) were significantly secreted by CD4 Th cell lines from 4 CRC patients when cocultured with PASD1-positive SW480 CRC cell lines. IL-17A is a proinflammatory cytokine, known for its function in regulating tumor progression in CRC (Zhao et al., 2020). It also plays a crucial role in tumor progression by increasing the tumor size. On the other hand, IL-6 is highly associated with the production of CD4 T cells (Sofi et al., 2009). Thus, IL-17A and IL-6 cytokines have an inverse correlation in tumor immunology. The coculture of CD4 Th cell lines from C5 with SW480 cell line showed a lower level of IL-17A because the SW480 cancer cells underwent apoptosis while high IL-6 was detected due to the high proliferation of CD4 Th cell lines from C5 which cause the cell cytotoxicity. This provides evidence of the mechanism of homeostasis between T cells and tumor cells.

## 5. Conclusion

This current study shows the highest PASD1 protein expression in the CRC population. This is the first report on CD4 Th response to a PASD1 by CRC and polyps patients and cell cytotoxicity to PASD1-positive cell CRC cell line. These CD4 Th peptides hold a future potential for use in conjunction with PASD1 peptides eliciting CTL in a vaccine for CRC and polyps patients’ immunotherapy. Some limitations of the study included samples size and insufficiency of matched FFPE tissue sections and blood samples due to priority having to be given to the diagnostic pathology.

## Supplementary

**Supplementary S1:** Demographic data of CRC patients. NA = Non-applicable, SD= standard deviation, IQR=Inter quartile range.

Demographic data	CRC ( *n* = 60)	Polyps ( *n* = 17)	Normal ( *n* = 13)
	(n, %)	(n, %)	(n, %)

**Age**	Mean = 65 (30–85 yo)	Mean = 67 (51–85 yo)	Mean = 64 (50 – 76 yo)
	Median = 66.5;	Median = 68;	Median = 64;
	SD = 11.6; IQR = 13.5	SD = 9.2; IQR = 12	SD = 7.5; IQR = 8
≤ 60 years old	19 (31.7%)	2 (11.8%)	4 (30.8%)
> 60 years old	41 (68.3%)	15 (88.2%)	9 (69.2%)

**Sex**			
Male	36 (60.0%)	12 (70.5%)	9 (69.2%)
Female	24 (40.0%)	5 (29.4%)	4 (30.8%)

**Ethnicity**			
Malay	28 (46.7%)	7 (41.2%)	5 (38.5%)
Chinese	28 (46.7%)	10 (58.8%)	7 (53.8%)
Indian	2 (3.3%)	0	1 (7.7%)
Others	2 (3.3%)	0	0

**Surgical resection**			
Yes	60 (100.0%)	NA	NA
No	0 (0.0%)		

**Tumor/polyps location**			
Distal	50 (83.3%)	11 (64.7%)	NA
Proximal	10 (16.7%)	6 (35.3%)	

**Types of the tumor /polyps**	Adenocarcinoma	Hyperplastic polyps	
	59 (98.3%)	12 (70.5%)	NA
	Neuroendocrine carcinoma	Tubulovillous adenoma	
		4 (23.5%)	
	1 (1.7%)		
		Tubular adenoma	
		1 (6.0%)	

**Histological grade**	Well-differentiated	(only applicable for adenomatous polyps)	NA
	33 (55.0%)		
	Moderately	High-grade dysplasia	
	Differentiated	1 (20.0%)	
	26 (43.3%)	Low-grade dysplasia	
	Poorly differentiated	4 (80.0%)	
	1 (1.7%)		

**Tumor stage (TNM)**			
T1	10 (16.7%)	NA	NA
T2	13 (21.6%)		
T3	25 (41.7%)		
T4	12 (20.0%)		

**Nodal metastasis**			
N0	23 (38.3%)	NA	NA
N1–2	37 (61.7%)		

**Distant metastasis**			
M0	48 (80.0%)	NA	NA
M1	12 (20.0%)		

**Supplementary S2:** Summary of PASD1 protein expression and clinical characteristics of CRC patients

Characteristics	Positive PASD1 protein expression in CRC patients	

	PASD1-1 and PASD1-2	PASD1-2 only
	( *n = 19*)	( *n = 2*)
**Age**		
≤ 60 years old	7/19 (36.8%)	1/2 (50.0%)
> 60 years old	12/19 (63.2%)	1/2 (50.0%)
**Sex**		
Male	7/19 (36.8%)	1/2 (50.0%)
Female	12/19 (63.2%)	1/2 (50.0%)
**Race**		
Malay	7/19 (36.8%)	1/2 (50.0%)
Chinese	11/19 (57.9%)	1/2 (50.0%)
Indian	0/19 (0.0%)	0/2 (0.0%)
Others	1/19 (5.3%)	0/2 (0.0%)
**Tumor location**		
Distal	16/19 (84.2%)	0/2 (0.0%)
Proximal	3/19 (15.8%)	2/2 (100.0%)
**Surgical resection**		
Yes	19/19 (100.0%)	2/2 (100.0%)
No	0/19 (0.0%)	0/2 (0.0%)
**Tumor stage (TNM)**		
T1	2/19 (10.5%)	0/2 (0.0%)
T2	5/19 (26.3%)	1/2 (50.0%)
T3	7/19 (36.8%)	1/2 (50.0%)
T4	5/19 (26.3%)	0/2 (0.0%)
**Nodal metastasis**		
N0	12/19 (63.2%)	1/2 (50.0%)
N1–2	7/19 (36.8%)	1/2 (50.0%)
**Distant metastasis**		
M0	14/19 (73.7%)	2/2 (100.0%)
M1	5/19 (26.3%)	0/2 (0.0%)
**Types of tumor**		
Adenocarcinoma	19/19 (100.0%)	2/2 (100.0%)
Neuroendocrine carcinoma	0/19 (0.0%)	0/2 (0.0%)
**Histological grade**		
Well-differentiated	13/19 (68.4%)	2/2 (100.0%)
Moderately differentiated	5/19 (26.3%)	0/2 (0.0%)
Poorly differentiated	1/19 (5.3%)	0/2 (0.0%)

**Supplementary S3:** Summary of PASD1 protein expressions with clinical data of polyp patients

Characteristics	Positive PASD1 protein expression in polyp patients	

	PASD1-1 and PASD1-2	PASD1-2 only
	( *n* = 5)	*(n* = 0 *)*
**Age**		
≤ 60 years old	1/5 (20.0%)	0/0 (0.0%)
> 60 years old	4/5 (80.0%)	0/0 (0.0%)
**Sex**		
Male	4/5 (80.0%)	0/0 (0.0%)
Female	1/5 (20.0%)	0/0 (0.0%)
**Ethnicity**		
Malay	0/5 (0.0%)	0/0 (0.0%)
Chinese	5/5 (100.0%)	0/0 (0.0%)
**Polyp location**		
Distal	4/5 (80.0%)	0/0 (0.0%)
Proximal	1/5 (20.0%)	0/0 (0.0%)
**Types of polyp**		
Hyperplastic polyp	5/5 (100.0%)	0/0 (0.0%)
Tubulovillous adenoma	0/5 (0.0%)	0/0 (0.0%)
Tubular adenoma	0/5 (0.0%)	0/0 (0.0%)
**Histological grade (only applicable for adenomatous polyps)**		
High-grade dysplasia	0/5 (0.0%)	0/0 (0.0%)
Low-grade dysplasia	0/5 (0.0%)	0/0 (0.0%)

## Figures and Tables

**Table 1:** List of peptides used in ELISpot assay.

Peptides	Epitopes (aa)	Sequence
CD4 PASD1 (1)	38–47	EVEQYGPQENVHMFVDSTYC
CD4 PASD1 (2)	167–175	QQQLVQQEQHLKEQQRQLRE
CD4 PASD1 (3)	63–71	PQDYIRLWQELSDSLGPVVQV
CD4 irCD4	n/a	DESFRKYTAFTIPSMNNETP

**Table 2:** Frequency of positive PASD1 mRNA detection in CRC and polyps samples.

PASD1 primer	*PASD1_v1* and *PASD1_v2* (n, %)	*PASD1_v1* only (n, %)	*PASD1_v2* only (n, %)
**CRC (** ***n*** ** = 50)**	14 (28.0%)	5 (10.0%)	23 (46.0%)
**Polyps (** ***n*** ** = 6)**	2 (33.3%)	2 (33.3%)	2 (33.3%)

**Table 3:** PASD1 protein expression in CRC samples.

		CRC		Polyps	
Classifications	Criteria	PASD1-1 expression (n, %)	PASD1-2 expression (n, %)	PASD1-1 expression (n, %)	PASD1-2 expression (n, %)
Negative (−)	< 5% of tumor cells	41 / 60 (68.3%)	58 / 60 (96.7%)	12 / 17 (70.6%)	17 / 17 (100%)
Weak positive (+)	6 to 25% of tumor cells	13 / 60 (21.7%)	2 / 60 (3.3%)	4 / 17 (23.5%)	0 / 17 (0.0%)
Moderate positive (++)	26 to 74% of tumor cells	6 / 60 (10.0%)	0 / 60 (0.0%)	1 / 17 (5.95%)	0 / 17 (0.0%)
Strong positive (+++)	> 75% of tumor cells	0 / 60 (0.0%)	0 / 60 (0.0%)	0 / 17 (0.0%)	0 / 17 (0.0%)

**Table 4:** Positive CD4 immune response towards PASD1 peptides in CRC and polyps sample. Quantification is through IFN-γ release assay ELISpot. C = cancer sample, P = polyps sample.

Sample ID	Interferon – γ release towards PASD1 peptides/50,000 cells (spots per well)					
	PASD1 (1)	PASD1 (2)	PASD1 (3)	Cells only	irCD4	PHA
C1	2 ± 2	5 ± 1	49 ± 10	4 ± 4	4 ± 1	216 ± 17
C5	9 ± 4	4 ± 2	3 ± 2	4 ± 4	2 ± 1	127 ± 13
C6	2 ± 1	6 ± 4	2 ± 1	2 ± 1	2 ± 1	136 ± 7
C9	7 ± 3	2 ± 1	2 ± 1	2 ± 1	2 ± 1	412 ± 15
C12	185 ± 9	5 ± 4	14 ± 8	13 ± 4	1 ± 4	325 ± 21
C16	9 ± 8	6 ± 2	7 ± 1	7 ± 2	3 ± 3	128 ± 14
C18	5 ± 3	4 ± 4	8 ± 2	5 ± 4	5 ± 1	276 ± 11
C19	4 ± 4	2 ± 1	9 ± 3	4 ± 1	6 ± 2	159 ± 10
C26	17 ± 6	2 ± 2	7 ± 5	6 ± 6	3 ± 1	120 ± 17
C27	9 ± 6	5 ± 3	7 ± 3	3 ± 2	2 ± 2	180 ± 8
C28	10 ± 8	3 ± 2	8 ± 4	7 ± 2	5 ± 3	131 ± 6
C32	78 ± 15	7 ± 3	26 ± 5	17 ± 7	4 ± 2	153 ± 7
C47	4 ± 4	3 ± 2	8 ± 3	3 ± 1	3 ± 2	110 ± 12
P6	6 ± 2	4 ± 1	10 ± 3	4 ± 1	3 ± 1	136 ± 9
P7	2 ± 1	3 ± 2	7 ± 3	2 ± 1	2 ± 1	150 ± 20
P8	8 ± 5	57 ± 10	6 ± 2	4 ± 1	7 ± 3	275 ± 17
P12	89 ± 22	6 ± 2	25 ± 6	16 ± 7	4 ± 1	143 ± 10

## Data Availability

All data analyzed during this study are included in this published article and its supplementary information files.

## References

[b1-turkjbiol-46-5-361] Ait-Tahar K Liggins AP Collins GP Campbell A Barnardo M 2009 Cytolytic T-cell response to the PASD1 cancer-testis antigen in patients with diffuse large B-cell lymphoma British Journal of Haematology 146 4 396 407 10.1111/j.1365-2141.2009.07761.x 19552722

[b2-turkjbiol-46-5-361] Ait-Tahar K Liggins AP Collins GP Campbell A Barnardo M 2011 CD4-positive T-helper cell responses to the PASD1 protein in patients with diffuse large B-cell lymphoma Haematologica 96 1 78 86 10.3324/haematol.2010.028241 20851862 PMC3012768

[b3-turkjbiol-46-5-361] Azizah AM Hashimah B Nirmal K Siti Zubaidah AR Puteri NA 2019 Malaysian National Cancer Registry Report 2012–2016 Malaysia Cancer Statistics, Data and Figure National Cancer Institute 5

[b4-turkjbiol-46-5-361] Bray F Ferlay J Soerjomataram I Siegel RL Torre LA 2018 Global cancer statistics 2018: GLOBOCAN estimates of incidence and mortality worldwide for 36 cancers in 185 countries A Cancer Journal for Clinicians 68 6 394 424 10.3322/caac.21492 30207593

[b5-turkjbiol-46-5-361] Bustamante-Lopez LA Nahas SC Nahas CSR Pinto RA Marques CFS 2019 Is there a difference between right-versus left-sided colon cancers? Does side make any difference in long-term follow-up? Arquivos Brasileiros de Cirurgia Digestiva 32 4 e1479 10.1590/0102-672020190001e1479 31859932 PMC6918732

[b6-turkjbiol-46-5-361] Caballero OL Chen YT 2009 Cancer/testis (CT) antigens: potential targets for immunotherapy Cancer Science 100 11 2014 2021 10.1111/j.1349-7006.2009.01303.x 19719775 PMC11158245

[b7-turkjbiol-46-5-361] Cooper CD Liggins AP Ait-Tahar K Roncador G Banham AH 2006 PASD1, a DLBCL-associated cancer testis antigen and candidate for lymphoma immunotherapy Leukemia 20 2172 2174 10.1038/sj.leu.2404424 17024112

[b8-turkjbiol-46-5-361] Doghman-Bouguerra M Finetti P Durand N Parise IZS Sbiera S 2020 Cancer-testis Antigen FATE1 Expression in Adrenocortical Tumors Is Associated with A Pervasive Autoimmune Response and Is A Marker of Malignancy in Adult, but Not Children ACC Cancers 12 3 689 10.3390/cancers12030689 32183347 PMC7140037

[b9-turkjbiol-46-5-361] Fleming M Ravula S Tatishchev SF Wang HL 2012 Colorectal carcinoma: Pathologic aspects Journal of Gastrointestinal Oncology 3 3 153 173 10.3978/j.issn.2078-6891.2012.030 22943008 PMC3418538

[b10-turkjbiol-46-5-361] Giunco S Petrara MR Bergamo F Del Bianco P Zanchetta M 2019 Immune senescence and immune activation in elderly colorectal cancer patients Aging 11 11 3864 3875 10.18632/aging.102022 31195370 PMC6594805

[b11-turkjbiol-46-5-361] Hagiwara Y Sieverling L Hanif F Anton J Dickinson ER 2016 Consequences of point mutations in melanoma-associated antigen 4 (MAGE-A4) protein: Insights from structural and biophysical studies Scienctific Reports 6 25182 10.1038/srep25182 PMC4848555 27121989

[b12-turkjbiol-46-5-361] Hanahan D Weinberg RA 2011 Hallmarks of cancer: the next generation Cell 144 5 646 674 10.1016/j.cell.2011.02.013 21376230

[b13-turkjbiol-46-5-361] Johdi NA Mohd Said HZ Mohd Idris MR Ismail NA Mustangin M 2019 PASD1 Expression in Malaysian Hematological Malignancies Patients Journal of Biochemistry Microbiology and Biotechnology 7 1 1 4 https://journal.hibiscuspublisher.com/index.php/JOBIMB/article/view/444

[b14-turkjbiol-46-5-361] Johdi NA Sukor NF 2020 Colorectal Cancer Immunotherapy: Options and Strategies Frontiers in Immunology 11 1624 10.3389/fimmu.2020.01624 33042104 PMC7530194

[b15-turkjbiol-46-5-361] Li R Guo M Song L 2019 PAS Domain Containing Repressor 1 (PASD1) Promotes Glioma Cell Proliferation Through Inhibiting Apoptosis In Vitro Medical Science Monitor 25 6955 6964 10.12659/MSM.916308 31558691 PMC6761850

[b16-turkjbiol-46-5-361] Liggins AP Brown PJ Asker K Pulford K Banham AH 2004 A novel diffuse large B-cell lymphoma-associated cancer testis antigen encoding a PAS domain protein British Journal of Cancer 91 1 141 149 10.1038/sj.bjc.6601875 15162151 PMC2364759

[b17-turkjbiol-46-5-361] Lian J Ma L Yang J Xu L 2015 Aberrant Gene Expression Profile of Unaffected Colon Mucosa from Patients with Unifocal Colon Polyp Medical Science Monitor 21 3935 3940 10.12659/MSM.895576 26675397 PMC4687947

[b18-turkjbiol-46-5-361] Lin JH Zhang SM Rexrode KM Manson JE Chan AT 2013 Association between sex hormones and colorectal cancer risk in men and women Clinical Gastroenterology Hepatology 11 4 419 424e411 10.1016/j.cgh.2012.11.012 23200979 PMC3594467

[b19-turkjbiol-46-5-361] Michael AK Harvey SL Sammons PJ Anderson AP Kopalle HM 2015 Cancer/Testis Antigen PASD1 Silences the Circadian Clock Molecular Cell 58 5 743 754 10.1016/j.molcel.2015.03.031 25936801 PMC4458219

[b20-turkjbiol-46-5-361] Pai RK Bettington M Srivastava A Rosty C 2019 An update on the morphology and molecular pathology of serrated colorectal polyps and associated carcinomas Modern Pathology 32 10 1390 1415 10.1038/s41379-019-0280-2 31028362

[b21-turkjbiol-46-5-361] Pan J Cen L Xu L Miao M Li Y 2020 Prevalence and risk factors for colorectal polyps in a Chinese population: a retrospective study Scientific Reports 10 1 6974 10.1038/s41598-020-63827-6 32332839 PMC7181769

[b22-turkjbiol-46-5-361] Rahmatallah Y Khaidakov M Lai KK Goyne HE Lamps LW 2017 Platform-independent gene expression signature differentiates sessile serrated adenomas/polyps and hyperplastic polyps of the colon BMC Medical Genomics 10 1 81 10.1186/s12920-017-0317-7 29284484 PMC5745747

[b23-turkjbiol-46-5-361] Scanlan MJ Simpson AJ Old LJ 2004 The cancer/testis genes: review, standardization, and commentary Cancer Immunology 4 1 14738373

[b24-turkjbiol-46-5-361] Schliemann D Paramasivam D Dahlui M Cardwell CR Somasundaram S 2020 Change in public awareness of colorectal cancer symptoms following the Be Cancer Alert Campaign in the multi-ethnic population of Malaysia BMC Cancer 20 1 252 10.1186/s12885-020-06742-3 32213173 PMC7093961

[b25-turkjbiol-46-5-361] Schirrmacher V 2019 From chemotherapy to biological therapy: A review of novel concepts to reduce the side effects of systemic cancer treatment (Review) International Journal of Oncology 54 2 407 419 10.3892/ijo.2018.4661 30570109 PMC6317661

[b26-turkjbiol-46-5-361] Sindhu CK Nijar AK Leong PY Li ZQ Hong CY 2019 Awareness of Colorectal Cancer among the Urban Population in the Klang Valley Malays Family Physician 14 3 18 27 32175037 PMC7067497

[b27-turkjbiol-46-5-361] Siripongpreeda B Mahidol C Dusitanond N Sriprayoon T Muyphuag B 2016 High prevalence of advanced colorectal neoplasia in the Thai population: a prospective screening colonoscopy of 1404 cases BMC Gastroenterology 16 1 101 10.1186/s12876-016-0526-0 27553627 PMC4995664

[b28-turkjbiol-46-5-361] Soh JE Abu N Sagap I Mazlan L Yahaya A 2019 Validation of immunogenic PASD1 peptides against HLA-A*24-02 colorectal cancer Immunotherapy 11 14 1205 1219 10.2217/imt-2019-0073 31478431

[b29-turkjbiol-46-5-361] Tan YJ Wendy T Chieng JY 2019 Detection rate of colonic polyp among patients who had undergone colonoscopy at gastroenterology unit of Serdang Hospital, Malaysia Medical Journal Malaysia 74 1 20 24 30846657

[b30-turkjbiol-46-5-361] Vijg J Dong X Milholland B Zhang L 2017 Genome instability: a conserved mechanism of ageing? Essays Biochemistry 61 1 305 315 10.1016/j.tma.2017.09.003 PMC5988260 28550046

[b31-turkjbiol-46-5-361] Yamada R Takahashi A Torigoe T Morita R Tamura Y 2013 Preferential expression of cancer/testis genes in cancer stem-like cells: proposal of a novel sub-category, cancer/testis/stem gene Tissue Antigens 81 6 428 434 10.1111/tan.12113 23574628

[b32-turkjbiol-46-5-361] Yang J Xiong LJ Xu F Zhao X Liu B 2013 Estrogen inhibits colon polyp formation by reducing angiogenesis in a carcinogen-induced rat model International Journal of Endocrinology 2013 453898 10.1155/2013/453898. PMC3848267 24348555

[b33-turkjbiol-46-5-361] Zhang Y Wang Z Zhang J Lim SH 2009 Core promoter sequence of SEMG I spans between the two putative GATA-1 binding domains and is responsive to IL-4 and IL-6 in myeloma cells Leukemia Research 33 1 166 169 10.1016/j.leukres.2008.05.021 18602691 PMC2605945

[b34-turkjbiol-46-5-361] Zhao J Chen X Herjan T Li X 2020 The role of interleukin-17 in tumor development and progression Journal Experimental Medicine 217 1 e20190297 10.1084/jem.20190297 PMC7037244 31727782

